# Programming Factors of Neonatal Intestinal Dysbiosis as a Cause of Disease

**DOI:** 10.3390/ijms24065723

**Published:** 2023-03-17

**Authors:** Miljana Z. Jovandaric, Stefan Dugalic, Sandra Babic, Ivana R. Babovic, Srboljub Milicevic, Dejan Mihajlovic, Miljan Culjic, Tamara Zivanovic, Aleksandar Trklja, Bogdan Markovic, Vera Plesinac, Zorica Jestrovic, Biljana Medjo, Misela Raus, Miroslava Gojnic Dugalic

**Affiliations:** 1 Department of Neonatology, Clinic for Gynecology and Obstetrics, University Clinical Center of Serbia, 11000 Belgrade, Serbia; 2 Department of Gynecology and Obstetrics, Clinic for Gynecology and Obstetrics, University Clinical Center of Serbia, 11000 Belgrade, Serbia; 3 Faculty of Medicine, University of Belgrade, 11000 Belgrade, Serbia; 4 Faculty of Medicine, University of Pristina Temporarily Settled in Kosovska Mitrovica, 38220, Serbia; 5 Department Pediatrics and Neonatal Intensive Care, University Children’s Hospital, 11000 Belgrade, Serbia; 6 Department of Neonatology, University Children’s Hospital, 11000 Belgrade, Serbia

**Keywords:** intestinal microbiota, dysbiosis, neonates, diseases, biomarkers of inflammation

## Abstract

The intestinal microbiota consists of trillions of bacteria, viruses, and fungi that achieve a perfect symbiosis with the host. They perform immunological, metabolic, and endocrine functions in the body. The microbiota is formed intrauterine. Dysbiosis is a microbiome disorder characterized by an imbalance in the composition of the microbiota, as well as changes in their functional and metabolic activities. The causes of dysbiosis include improper nutrition in pregnant women, hormone therapy, the use of drugs, especially antibiotics, and a lack of exposure to the mother’s vaginal microbiota during natural birth. Changes in the intestinal microbiota are increasingly being identified in various diseases, starting in the early neonatal period into the adult period. Conclusions: In recent years, it has become more and more obvious that the components of the intestinal microbiota are crucial for the proper development of the immune system, and its disruption leads to disease.

## 1. Introduction

The human microbiota consists of different types of microorganisms that include bacteria, viruses, and fungi. They achieve symbiosis with the host and have important metabolic, immunological, and endocrine functions in the human body. The composition of the gut microbiota differs between individuals, varies during growth, and depends on host-environment interactions. The gut microbiota is constantly exposed to various external influences [1]. In the intestinal tract, bacteria, viruses, and fungi are in dynamic balance. The existence of viruses as part of the intestinal microbiome has been neglected for a long time; 90% of them are in the form of bacteriophages, and approximately 10% of viruses are plant or animal viruses that are ingested with food. A community of bacteria, viruses, and fungi colonize the intestinal tract before birth, contributing to nutrient metabolism, stimulating the immune system, and protecting the host from pathogens [2]. 

By comparing the intestinal microbiota of full-term and premature infants, a significant difference in the colonization of the intestinal microbiota was determined. *Bacteroides* is present in full-term infants in the first year, *Parabacteroides* and *Christensenellaceae* in the second year, and *Lactobacillus, Streptococcus* (second year), and *Carnobacterium* (fourth year) in premature infants in the first year. These differences disappear after the fourth year of life [3].

The violation of this balance, when the “good bacteria” are unable to control the “bad” bacteria, is called dysbiosis. Dysbiosis of the intestinal tract is associated with pathology in the early neonatal period, as well as in adulthood (gluten enteropathy, intolerance to food ingredients, allergies, asthma, metabolic disorders, autism, cancers, and many other autoimmune diseases) [4]. Newborns adapt to the extrauterine environmental conditions by developing intestinal immunological homeostasis. In order to achieve the adequate development of intestinal immunological homeostasis, appropriate bacterial colonization is needed. The most important factor for proper colonization is breastfeeding, as close contact with the mother’s breast provides emotional security to the newborn, especially those who are sick and prematurely born. In addition, breast milk contains various substances, including simple sugars and complex hormones, which, due to their pronounced biological activity in the infant, are called the bioactive complex of breast milk. Human milk mostly consists of water and nutrients dissolved in it: proteins, fats, carbohydrates, vitamins, minerals, and trace elements. It is known that there are other factors with specific immune functions. The discovery of the mother’s broncho-mammary and intramammary circulation showed that the pathogenic microorganisms that endanger the mother through the digestive or respiratory tract produce specific antibodies in the mother’s milk. The mother’s milk also contains virus fragments without the ability to reproduce, but are antigenically potent enough to cause the creation of specific antibodies in the infant. In this way, they participate in the maturation of the infant’s immune system [5].

**Figure 1:**
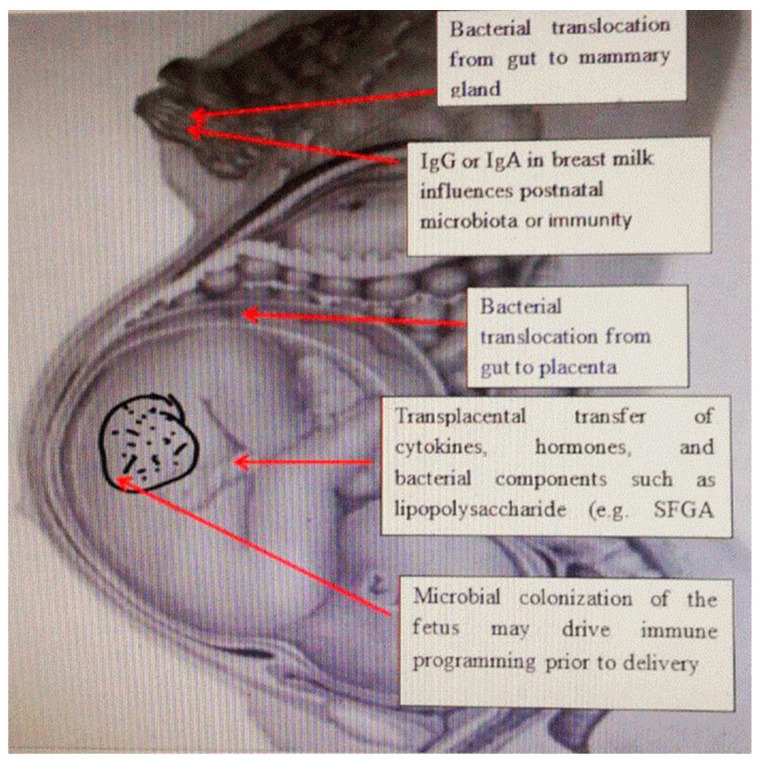
The relationship between maternal microbiota and newborn immunity. Commensal microbes from the intestine of the pregnant woman, and the placenta and mammary glands affect the development of immunity in the fetus and the newborn by releasing short-chain fatty acids, and antibodies and changing the cytokine environment.

The formation and multiplication of the gut microbiome start from birth, while the modification of their composition depends mainly on various genetic, nutritional, and environmental factors. The primary obstacle to the proper development of intestinal microbiota is improper nutrition (excessive consumption of processed food of animal origin, insufficient intake of fresh fruit and especially vegetables, as well as vegetable juices, unnecessary overuse of antibiotics, and insufficient intake of breast milk or a short period of breastfeeding), which increases the inflammatory potential of intestinal microbiota [6]. The microbiota of the intestine, vagina, and breast milk influence the colonization of newborns. The composition of the mother’s gut before delivery affects the creation of fetal immunity. Although the function of the placental microbiome is not clear, it is known that the gut microbiota during pregnancy is the most important factor in the health of the offspring [7]. The passage of bacteria from the intestines of pregnant women and its colonization in extraintestinal places during pregnancy explains the presence of bacteria in breast milk. The transfer of antibodies through breastfeeding provides immune protection to the newborn; however, IgG-related bacteria can also be transferred after birth, as well as the transplacental IgG-mediated transfer of bacteria that can affect the developing immune system. This suggests that fetal immune programming in utero depends in part on the IgG-mediated transfer of bacterial components. Transplacental immune regulation can also be mediated by cytokines and hormones, as well as bacterial components such as lipopolysaccharides [8] (Figure 1).

## 2. Intestinal Microbiome of Newborns

The gastrointestinal tract (GI) is undoubtedly the largest and most important organ of the immune system in the body, having a central role in the immune response of the organism. The intestinal epithelial barrier is in constant interaction with the intestinal microbiome and the cells of the immune system. The communication between epithelial cells, immune cells, and the microbiome is the most important as the specific immune responses to antigens depend on this communication. A change in the intestinal microorganisms (dysbiosis) induces the immune response and increases the body’s sensitivity to most diseases in early and adult life [9].

Upon birth, the intestinal microbiota of the newborn is dominated by the genera *Enterobacteriaceae* and *Staphylococcus*. Following that, lactic acid bacteria and *Bifidobacterium* are dominant. Bacteria from the genus *Bifidobacterium* prevail until the introduction of solid food. After the weaning period, the *Bacteroides, Prevotella, Ruminococcus, Clostridium*, and *Veillonella* genera dominate. The microbiota prior to the weaning period is rich in the bacteria that facilitate the use of lactate, while after the weaning period, solid food promotes the growth of bacteria that contain genes that code for carbohydrate breakdown, vitamin synthesis, and xenobiotic degradation [10]. The type of birth is one of the factors that influence the development of the microbiota in early childhood. In children born vaginally, the gut microbiota is similar to the mother’s vaginal microbiota, where *Lactobacillus, Prevotella*, and *Sneathia* genera predominate, while the gut microbiota of children born by cesarean section is similar to the skin microbiota, where *Staphylococcus, Corynebacterium* and *Propionibacterium* genera predominate. Some studies have shown that the colonization by the *Bacteroides* and *Bifidobacterium* genera starts one month after birth, while the concentration of *Clostridium difficile* is very high. A decrease in the presence of Bacteroides after three to four months in children born by cesarean section has also been reported, and the microbiota of these children was less diverse [11].

The gut microbiota develops intensively from the second to the third year of life. After the third year of life, it is similar to the microbiota of an adult. The establishment of stable gut microbiota is accompanied by two important changes in childhood. The first occurs shortly after birth, during breastfeeding, when the dominant microbes are the genus Bifidobacterium. Another change occurs during the period of introducing solid food into the child’s diet. In this period, bacteria from the genus *Bacteroides* and *Firmicutes* dominate [12].

Between approximately 5 and 18% of newborns are born prematurely, and 0.5% are children with extremely small body weights and with <25 gestational weeks (GW) [3]. The intestinal microbiota of newborns with very low birth weight is made up of an abundance of bacilli from gammaproteobacteria to clostridia [13]. Children who developed early NEC (within ten days of life) showed a predominance of *Firmicutes*. Various bacteria from the class *Clostridia* have also been associated with early NEC. Along with this increase in *Firmicutes,* a decrease in *Gammaproteobacteria* was observed in these early cases. Late-onset NEC (after ten days of life) was associated with an increase in *Gammaproteobacteria* with a concomitant decrease in *Firmicutes* (especially *Negativicutes*) [14,15]. Children who received antibiotics showed an increased relative amount of Proteobacteria and a decreased relative abundance of *Firmicutes* and *Actinobacteria*. This may explain the increased risk of NEC after antibiotic administration in preterm infants. The mode of delivery leads to differences in microbiota, but these were not associated with NEC. The relative abundance of *Firmicutes* was higher after cesarean section, and that of *Bacteroides* was higher after vaginal delivery. Formula-fed infants showed higher amounts of *Firmicutes* and breast-fed infants showed higher relative amounts of *Proteobacteria*, but this was not associated with the development of NEC [16]. Preterm infants receiving breast milk show less interindividual variance in microbiota compared to formula-fed infants [17]. Breastfed preterm infants have a lower risk of NEC; this may be based on the fact that, in addition to breast milk containing specific macronutrients, polyunsaturated fatty acids, lactoferrin, immune cells, and immunoglobulins, it also contains microbes that appear to be personalized for each maternal pair-child [18]. 

A healthy intestinal flora is essential for the health of the host. The normal human microbiome consists of two bacterial phyla, *Bacteroidetes* and *Firmicutes*. The intestinal microbiota of neonates is developed by the end of the third year of life. The total number of bacteria is significantly lower in newborns than in adults and the elderly. In the fecal microbiota of newborns, bifidobacteria are the dominant microbial group. The number of *Firmicutes/Bacteroidetes* increases in later life and continues to change with age [19]. The *Firmicutes/Bacteroidetes* ratio (F/B ratio) is an indicator of inflammatory bowel disease [4]. Prematurely born children of pregnant women who received antibiotics have an increase in *Enterobacteriaceae* (microorganisms belonging to the type *Proteobacteria*), as well as a decrease in the amount of the *Bacteroidaceae* family [20].

Normal intestinal microbiota participates in the proper nutrient and drug metabolism, maintaining the structural integrity of the intestinal mucosa barrier, immunomodulation, and protection against pathogens [21].

The factors that change the intestinal microbiota are diverse and act in different periods of life. They include the method of delivery (vaginal or cesarean section); nutrition after birth (mother’s milk or adapted food); nutrition later in life (vegan, vegetarian, or diet including large quantities of meat or meat products); as well as antibiotics or molecules similar to the antibiotics that most often originate from food [22]. The mother’s diet during pregnancy has a great influence on the formation of the intestinal microbiota of neonates. A diet rich in dietary fibers and proteins affects the creation of short-chain fatty acids (SCFA), which are known to be the main metabolites produced by the gut microbiota [23].

The intestines and brain are the boundaries of newborns with the fastest changes during intrauterine development and the postnatal period, and disturbances in the colonization process can cause consequences in behavioral disorders, as well as disturbances in the cognitive functions of newborns, motor activities, anxiety, and social behavior disorders [24,25].

Short-chain fatty acids play a role in the development of intestinal immunity by stimulating the development of memory CD4+ and CD8+ lymphocyte T cells and preserving the integrity of the intestinal epithelium [26]. In addition, bacterial metabolites are potentially transferred from the maternal gut during pregnancy to the mammary glands and may influence postnatal immune development during breastfeeding. Butyric acid stimulates the expression of brain-derived neurotrophic factor (BDNF), which is involved in neurogenesis [27]. Propionic acid has an impact on brain development, cognition, and behavior [28].

### The Role of Bacteria from Breast Milk in the Intestines of Newborns

Breast milk bacteria influence the frequency and severity of infections in breastfed infants through various mechanisms of antimicrobial metabolite production or by improving the intestinal barrier through increased mucin secretion and decreased permeability. The administration of a *Lactobacillus* strain isolated from breast milk to infants over six months resulted in a 46% reduction in gastrointestinal infections, 27% in upper respiratory tract infections, and 30% in other infections [29].

*Coagulase-negative staphylococci* and viridans streptococci may be particularly useful in reducing the unwanted pathogens to which infants are exposed in the hospital environment. Some *Staphilococcus epidermidis* strains that inhibit the in vivo colonization of *Staphylococcus aureus* have been proposed as a future strategy to eradicate this pathogen from the mucosal surfaces. Similarly, Viridans group streptococci (VGS) have been shown to inhibit the oral colonization of methicillin-resistant *Staphylococcus aureus* (MRSA) in infants exposed to the hospital environment. The bacteria in breast milk also affect the proper maturation of the immune system. Their function has a certain level of flexibility, depending on the conditions found in the intestinal environment. For example, *Lactobacillus salivarius* and *Lactobacillus fermentum* enhance the Th1 macrophage production of cytokines, such as IL-2 and IL-12, and the inflammatory mediator TNF-alpha, in the absence of inflammatory stimuli. However, both strains led to a reduction in Th1 cytokines when the cells were incubated in the presence of lipopolysaccharide [30,31].

Breast milk bacteria have a metabolic role in infants. The glycome of some lactobacilli and bifidobacteria can help create healthy intestinal microbiota in infants. The same microorganisms play a role in the breakdown of sugar and protein, and it should be emphasized that the passage of food through a child’s digestive tract is shorter than in adults and that the pH of the stomach is higher in infants. In this context, the lactobacilli strains from breast milk are metabolically active in the intestines of infants and increase the production of functional metabolites, such as butyrate, which is the main source of energy for colonocytes and an important factor in establishing the functionality of the digestive system. As a result, they improve bowel function by increasing the number, consistency, and volume of bowel movements [32].

## 3. Impact of Antibiotics on Gut Microbiota

Considering the disruption of intestinal microbiota in infants who received antibiotics, the question arises: at which age antibiotics are the oldest? Several studies have investigated how antibiotics work when given immediately after delivery (to the child) or before delivery (to the mother) [33].

In a study of 15 premature infants, a short treatment (≤3 days) with antibiotics led to a reduction in bifidobacteria in the gut of the infants immediately after treatment until the third week after birth [34]. 

Long treatment (≥5 days) caused the number of bifidobacteria to remain reduced until the sixth postnatal week. Children who received only short-term antibiotics had a gut microbiota that was similar to that of healthy babies, and their microbiota eventually recovered despite the initial changes in composition. In conclusion, the duration of antibiotic use was the main factor responsible for the change in microbiota [35,36,37]. In early neonatal life, the bacterial community of infants of intrapartum antibiotic prophylaxis (IAP) mothers for Group B *Streptococcus* (GBS) differed from unexposed infants [38]. Within an hour of antibiotic administration during natural childbirth, there was a 7.2% reduction in *bifidobacteria* and a positive growth of *clostridia*. In addition, there were low numbers of *Actinobacteria* and *Bacteroidetes*, as well as an overabundance of *Proteobacteria* [39].

The type and duration of antibiotic administration during pregnancy change the bacterial composition of the amniotic fluid, as well as the vagina, which can affect the microbiological composition of the baby at birth [40]. 

The number of babies born by cesarean section (SC) has increased significantly in recent years. In developed countries, they account for over 27% of births, while in underdeveloped countries, it exceeds 50%. Antibiotics are given in one dose before CS, most often Penicillin; in cases of the mother being allergic to Penicillin, a cephalosporin antibiotic is given. Newborns born through CS have delayed intestinal colonization compared to children born vaginally because there is no contact with the vaginal and fecal microbiota of the mother and the skin of the perineum. In addition, they have reduced microbial diversity, which can last until the second year of life. In neonates born through SC in developed countries, a delay or absence of *Bacteroides* has been registered, while in developing countries, the early colonization of *Bacteroides* is attributed to the fecal bacteria in the surrounding environment. Premature children exposed to antibiotics have a reduced number of *Bacteroides* and *Bifidobacterium* bacteria [41]. The use of antibiotics intrapartum or in the early neonatal period, as well as the infections themselves, also affect the acquisition of microbiota. The use of antibiotics reduces the biodiversity of the microbiota and increases the growth of potentially pathogenic bacteria, which are resistant to antibiotics. However, it seems that these microbiota changes are not permanent. When the fecal samples of infants exposed to antibiotics were compared with the fecal samples of the same children after infancy, despite this previous use of antibiotics, the overall resistance of the microbiota to antibiotics was reduced and there was an increased diversity of microorganisms. This suggests that the changes in the microbiota caused by the use of antibiotics are not always permanent [42]. Exposure to antibiotics, as well as to infections, during the first six months of life, affects excessive weight gain and obesity in later childhood. Antibiotics such as vancomycin, often used in *Staphylococcus* positive and coagulase-negative sepsis, have a greater effect on increasing body fat than penicillin antibiotics. The use of antibiotics at birth has a greater impact than later in life. More importantly, even after the antibiotic-induced dysbiosis was regulated, the exposed mice had a metabolically obese phenotype. Considering the current knowledge, in addition to the usual preventive measures, reducing the use of antibiotics in early childhood should be underlined and pregnant women should be encouraged to have a vaginal birth and breastfeed when possible [43].

The SARS-CoV-2 virus infects all age groups, including infants and pregnant women. The placental tissue of pregnant women with COVID-19 infection is associated with histological changes, which may compromise the fetal environment, and this may later lead to adverse neonatal outcomes [44]. Despite the viral nature of the disease, antibiotics are often prescribed to patients with COVID-19, mainly because of suspected bacterial coinfection [45]. The prevalence of bacterial coinfection and secondary infection in hospitalized patients with COVID-19 is relatively low, ranging between 3.5% and 14.3% [46]. Recognizing bacterial co-infections in pregnant women with COVID-19 is crucial for adequate treatment, reducing the routine use of antibiotics, and minimizing the negative consequences of excessive use, primarily the development of antimicrobial resistance (AMR) [47]. During the COVID-19 pandemic, the use of multiple antibiotics together, as well as the administration of ineffective antimicrobial drugs, led to an increase in drug-resistant infections. As a result, multidrug resistance (MDR) among microorganisms to conventional antimicrobial agents is a consequence that humanity is yet to face [48]. The irrational use of antibiotics from the carbapenem group has led to resistance in the *Klebsiella pneumoniae* that produce β-lactamase (ESBL), *Enterobacterales* that produce carbapenem-resistant New Delhi metallo-β-lactamase (NDM), *Acinetobacter baumannii*, and methicillin-resistant *Staphylococcus aureus* (MRSA), *Staphylococcus aureus*, *Enterococcus* faecium, *Klebsiella pneumoniae, Pseudomonas aeruginosa, Acinetobacter baumanni* and *Enterobacter species* [49]. The mechanisms of antibiotic resistance are enzymatic destruction or the inactivation of the drug, an alteration of the target enzyme, alteration of the permeability of cell membranes, alteration of the ribosome structure, and alteration of the metabolic pathway [50]. Plasmids and transposons are responsible for the rapid development of drug resistance, as well as the transfer of resistance between different strains of bacteria [51].

## 4. Consequences of the Use of Antibiotics on Diseases of Neonates and Adult Diseases

The effects of antibiotics on the host by changing the intestinal microbiome are enormous and have an impact on various functions of the body, including changes in the immune system and metabolic activities, creating the basis for the development of many diseases in both the neonatal and infant periods, as well as in adulthood [52].

**Figure 2:**
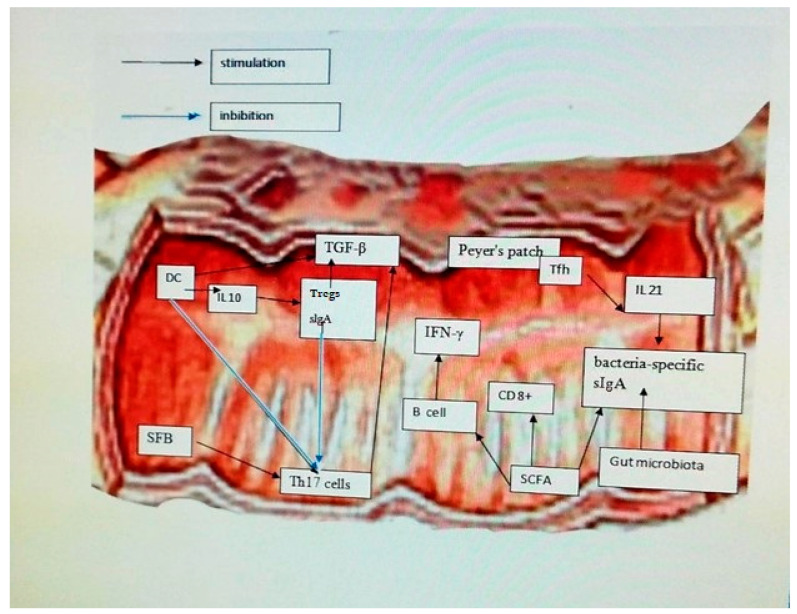
Effect of the modulatory role of intestinal microbiota and interactions between immune cells. Dendritic cells (DC) respond to microbes by secreting cytokines that trigger inflammation and stimulate the adaptive immune response by producing Tregs and sIgA by secreting IL-10. Parts of bacteria such as short-chain fatty acids (SCFA) induce T lymphocytes and B lymphocytes to produce gamma interferon (IFN-γ) from CD8+ T cells. Microbiota, SCFA, and IL-21 secreted from T follicular helper cells (Tfh) in Peyer’s patches (PP) contribute to the secretion of bacteria-specific sIgA. Sequestered fragments of bacteria (SFB) induce the production of Th17 cells. Tregs modulate the anti-inflammatory action of Transforming Growth Factor-β (TGF-β)-mediated DC and T helper 17 cells (Th17) e. DC and sIgA negatively regulate the pro-inflammatory function of Th17 cells by down-regulating synthesis 17 (IL17).

Trillions of bacteria inhabit the gastrointestinal (GI) tract, and the main role is played by commensals, which protect against the unwanted effects of pathogens. *Clostridium difficile* is a bacterium that occurs most often in hospitalized patients treated with antibiotics in the form of long-term, debilitating diarrhea that causes dehydration of the patient. The therapy of choice is the transfer of healthy microbiota to treat patients with recurrent infections. In addition, commensal bacteria *C. scindens* can inhibit the growth of *C. difficile* by producing bile acids, deoxycholic acid (DCA), and lithocholic acid (LCA) [38]. Dendritic cells (DC) respond to microbes by secreting cytokines that trigger inflammation and stimulate the adaptive immune response by producing Tregs and sIgA by secreting IL-10. Parts of bacteria, such as short-chain fatty acids (SCFA), induce T lymphocytes and B lymphocytes to produce gamma interferon (IFN-γ) from CD8+ T cells. Microbiota, SCFA, and IL-21 secreted from T follicular helper cells (Tfh) in Peyer’s patches (PP) contribute to the secretion of bacteria-specific sIgA. Sequestered fragments of bacteria (SFB) induce the production of Th17 cells. Tregs modulate the anti-inflammatory action of Transforming Growth Factor-β (TGF-β)-mediated DC and T helper 17 cells (Th17). DC and sIgA negatively regulate the pro-inflammatory function of Th17 cells by down-regulating synthesis 17 (IL17) [53] (Figure 2).

The consumption of food with artificial sweeteners leads to the deregulation of the intestinal microbiota, with an altered capacity to break down the glycans that affect reduced glucose tolerance, which is a pre-diabetes condition. Obese people have an altered microbiota enriched with clostridia that produce large amounts of DCA, contributing to liver inflammation and increasing the risk of developing liver cancer [54,55].

The pervasive developmental disorder includes a group of neurodevelopmental disorders of unknown etiology. Autism, which belongs to the group of pervasive developmental disorders, is the most common disorder from that group and appears no later than the third year of a child’s life. Genetic and environmental factors are considered to play an important role in the etiopathogenesis of the disease. This group of disorders is linked by characteristics such as difficulties in communication and social interaction and the presence of stereotyped actions. They are present in all patients and can be more or less pronounced. Children who have pervasive developmental disorders often also have symptoms of digestive system diseases, which indicates a possible role of the microbiota in the development of these disorders via the microbiota-intestine-brain axis. The increased permeability of the intestinal mucosa results in the presence of cytokines in the blood, such as interleukin-1b, interleukin-6, and interferon-g, but also parts of bacteria such as the lipopolysaccharide of gram-negative bacteria. By crossing the blood-brain barrier, they can activate an immune response in the brain and cause some of the behavioral symptoms of this spectrum of disorders. Differences in the microbiota of children with pervasive developmental disabilities compared to children without them exist. Children with this disorder have a less diverse microbiota, a smaller amount of *Bifidobacterium* spp. and *Firmicutes* spp., and higher levels of *Lactobacillus, Clostridium*, and *Bacteroidetes*. Despite this knowledge, a specific pattern of microbiota changes that could be used in the diagnosis or monitoring of individuals with pervasive developmental disorders has not yet been determined. Animal models have shown that mice born by cesarean section more often show signs of anxiety, worse social functioning, and repetitive actions, which has been confirmed by some human studies. In addition, their microbiota is disrupted and they show signs of increased intestinal permeability, which is a common comorbidity of pervasive developmental disorders [56].

One of the biggest public health problems today is obesity. It is thought that the prevalence of obesity will be 20% by 2025, which means that more than a billion people will be directly at risk from the metabolic consequences of obesity. The risk group among obese people is children, in whom the prevalence of obesity is growing at the same rate as in adults. Obesity is accompanied by complications such as type 2 diabetes, arterial hypertension, non-alcoholic fatty liver disease, metabolic syndrome, and many other conditions. Recent research focuses on the microbiota as a factor in the etiopathogenesis of the development of obesity [57]. 

Allergic diseases are increasingly prevalent in children and can persist as a problem even in adulthood. Improvements in hygiene and changes in diet are considered to be the main cause of the increase in the frequency of allergic diseases. This hypothesis is called the “hygiene hypothesis” and is thought to also influence changes in the development of the microbiota. The microbiota affects the host’s immune response, including the development of the immune system at an early age, and because the pathogenesis of allergic diseases is directly related to the immune system, the role of the microbiota as a cofactor in the development of allergic diseases is obvious [58]. Intestinal microorganisms, also in addition to all other microorganisms with which the newborn comes in contact, direct the development of this predominantly Th2 immune response into an immune response, which is more similar to adults and the maturation of regulatory T lymphocytes. This “immature” Th2 immune response causes an increased production of immunoglobulin E to various external antigens and the development of allergic reactions. By disrupting the normal development of the establishment of the intestinal microbiota, there is an increased risk for the development of allergic diseases, such as eczema, allergic rhinitis, and asthma. The use of antibiotics causes changes in the microbiota in children that create a predisposition to the development of allergic diseases. Taking antibiotics in the first year of life is associated with an increased frequency of asthma, as well as more severe forms. Stronger symptoms of rhinoconjunctivitis and eczema at ages six and seven are also associated with receiving antibiotics in infancy [59]. Newborns born by caesarean section have a different intestinal microbiota compared to children born vaginally. It is interesting that elective caesarean section carries a higher risk of developing allergic diseases than emergency caesarean section, which is explained by the fact that, in most emergency caesarean sections, the rupture of the amniotic membrane occurs prior to the decision to have the procedure. It is believed that breastfeeding protects the child from the development of allergic diseases with its probiotic and prebiotic components [60].

A change in the composition of the microbiota occurs before the development of the symptoms of allergic diseases, which could be used for the purpose of early diagnosis in the future. *Clostridium difficile* was found in greater numbers in children who later developed allergic diseases. For the development of allergic diseases, eczema, and asthma, the fact that there is dysbiosis is more important than the composition of the microbiota itself. These findings open up the possibility of early intervention and prevention of the development of allergic diseases in infants with increased risk. It has been shown that the use of preparations containing *Lactobacillus rhamnosus* GG (LGG) and *Lactobacillus fermentum* during pregnancy and in the early life of the child leads to a reduction in the risk of allergic dermatitis and a reduction in the severity of the symptoms of the disease [61].

### Biomarkers of Inflammation Associated with Gut Microbiota as a Potential for Giagnosing Diseases 

Neutrophils or polymorphonuclears are the most numerous leukocytes in the blood, their creation is stimulated by removed CSF (Colony stimulating factors), and the amount increases in response to some bacterial and fungal infections [62]. CRP is not only a marker of infection and inflammation, but also has a protective role against bacterial infections by activating the complementary and opsonizing bacteria. Although it is not a specific marker, elevated values are found in all inflammatory conditions, as well as infections. Its clinical importance is reflected in its easy performance and clinical variability in all population groups [63]. A study performed in patients with IBD, ulcerative colitis (UC), and Crohn’s disease (CD) showed that the serum calprotectin levels are directly related to the fecal calprotectin levels and are a more accurate diagnostic parameter of IBD compared to CRP. This study also showed that the combination of serum calprotectin with CRP or albumin may be helpful in predicting treatment escalation, especially in patients with CD. In patients with IBD who are in clinical remission, the measurement of the FC concentration can be used as a biological marker for the presence of inflammation of the mucous membrane [64,65]. The fecal calprotectin concentration reflects the degree of the pathological neutrophilic infiltration of the intestinal wall. As this is a non-invasive test, it has become popular in clinical practice for monitoring disease activity and adjusting anti-inflammatory treatment. Multicenter prospective clinical studies with larger numbers of patients and a separation of UC from the CD population are needed to define the exact role of FC in IBD diagnostic and therapeutic algorithms [66]. Zonulin is a human protein that increases permeability in the epithelial layer of the small intestine. Its proper functioning is crucial for the maintenance of the physiological processes in the intestines [67]. In the first study that included patients over 18 years of age, by Szymanska et al. [68], increased serum zonulin concentrations were found in various immunopathological diseases, such as food allergies, gastrointestinal tract infections, systemic autoimmune diseases, and inflammatory bowel diseases, although there are discrepancies in the concentration of zonulin in plasma and the concentration of fecal zonulin [69]. The limitation of zonulin as a parameter of intestinal inflammation is the small number of studies examining the neonatological population. In the study by Sochaczewska D et al. [70], the concentration of zonulin, occludin, and lipopolysaccharide in the stool of newborns was determined as the parameter of dysbiosis and intestinal permeability. The limitations of the study are that the results depend on the method of delivery, the intake of probiotics, or the diet of the mother, so this marker cannot currently be used in the diagnostics of inflammatory bowel diseases [71]. In inflammatory bowel disease, the concentration of leptin increases, while the level of adiponectin decreases. Resistin, produced by the immune system, is elevated in chronic colitis. The most significant role is played by fecal calprotectin as a marker of intestinal inflammation of necrotizing enterocolitis in premature children [72]. Myeloperoxidase (MPO) is a neutrophil enzyme that belongs to the peroxidase enzyme family and has bactericidal activity. MPO-deficient neutrophils perform the function of phagocytosis, but require the activity of the MPO enzyme when it comes to fungal infections. In a study by Rodríguez-Benítez MV et al. [73], MPO from meconium showed a correlation with fecal calpotectin in preterm infants with necrotic enterocolitis [74].

## 5. Therapeutic Directions of Treatment of Intestinal Dysbiosis

The development of the gastrointestinal microbiota of the fetus begins during intrauterine life, and a disturbed microbial balance in the mother’s gastrointestinal tract can be the cause of dysbiosis in the unborn child. The composition of the intestinal microbiota determines the appropriate type of immune response and the strength of intercellular connections in the intestinal epithelium. One of the basic types of intercellular junctions within the intestine are tight junctions (TJs). TJs are multiprotein complexes of integral membrane proteins (claudins and occludins) and cytoplasmic membrane proteins [75].

They are the most important regulatory element of intestinal permeability and maintain cell polarity by limiting the movement of proteins across the cell membrane. Disrupted intestinal colonization can damage the intestinal barrier by impairing the expression and function of TJ-building proteins, resulting in the loosening of the intercellular junctions. There is evidence to suggest that the disruption of the intestinal epithelial barrier increases the movement of bacteria and bacteria-associated products across the epithelium [76].

As a consequence, dysbiosis in the early neonatal period can result in the so-called leaky gut syndrome and promote the development of food allergies, recurrent infections, and autoimmune diseases, including irritable bowel syndrome, Hashimoto’s disease, obesity, asthma, and diabetes [77]. According to recent reports, the reduction in TJ expression in some neurodegenerative diseases (e.g., Parkinson’s disease) is associated with increased intestinal permeability, i.e., leaky gut syndrome [78].

It is necessary to use probiotic drops during the newborn period in order to reduce the immune response and achieve the balance of the Th1/Th2 subpopulation of T lymphocytes [26]. Microbiome-based interventions have traditionally focused on probiotics, usually lactobacilli and bifidobacteria, or prebiotics with non-digestible oligosaccharides [79].

Determining the genome is more important than the representation of the bacterial species. At present, there is increasing evidence that diseases are not triggered by the abundance of individual bacterial populations, but rather, are triggered by the collective microbiome (i.e., microbial consortia of functional genes and pathways) and its metabolites, which are called “functional core microbiome”. Clinically, the discovery of how we can alter the “functional core of the microbiome” to prevent or treat disease still has a long way to go before being put into practice [80].

## 6. Conclusions

The intestinal microbiota of a newborn is extremely prone to changes; therefore, its composition is influenced by the method of delivery, diet, use of antibiotics and probiotics by the mother, environment, socioeconomic status of the mother, geographical location, infections with pathogenic microorganisms, and many other factors. All of these factors can lead to dysbiosis, which creates predispositions for the development of disease from childhood to adulthood. This period of the newborn’s life could be crucial for interventions on the intestinal microbiota in order to reduce the risks of developing chronic diseases by correcting dysbiosis.

## Data Availability

All of the data are available in the archives (database) of Medline and PubMed.

## References

[1] Adak A., Khan M.R. (2018). An insight into gut microbiota and its functionalities. Cell. Mol. Life Sci..

[2] Butel M.-J., Waligora-Dupriet A.-J., Wydau-Dematteis S. (2018). The developing gut microbiota and its consequences for health. J. Dev. Orig. Health Dis..

[3] Fouhy F., Watkins C., Hill C.J., O’Shea C.-A., Nagle B., Dempsey E.M., O’Toole P.W., Ross R., Ryan C.A., Stanton C. (2019). Perinatal factors affect the gut microbiota up to four years after birth. Nat. Commun..

[4] Gagliardi A., Totino V., Cacciotti F., Iebba V., Neroni B., Bonfiglio G., Trancassini M., Passariello C., Pantanella F., Schippa S. (2018). Rebuilding the Gut Microbiota Ecosystem. Int. J. Environ. Res. Public Health.

[5] Konturek P., Konturek K., Zopf Y., Harsch I.A. (2020). Intestinal microbiota—A vital “organ” with manifold functions. MMW-Fortschritte der Medizin.

[6] Beam A., Clinger E., Hao L. (2021). Effect of Diet and Dietary Components on the Composition of the Gut Microbiota. Nutrients.

[7] Collado M.C., Rautava S., Aakko J., Isolauri E., Salminen S. (2016). Human gut colonisation may be initiated in utero by distinct microbial communities in the placenta and amniotic fluid. Sci. Rep..

[8] Nyangahu D.D., Jaspan H.B. (2019). Influence of maternal microbiota during pregnancy on infant immunity. Clin. Exp. Immunol..

[9] Fülöp V., Demeter J., Cseh Á. (2021). Significance and effects of prenatal and postnatal microbiome in the period of early individual development and options for interventional treatment. Orv. Hetil..

[10] Patangia D.V., Ryan C.A., Dempsey E., Ross R.P., Stanton C. (2022). Impact of antibiotics on the human microbiome and consequences for host health. Microbiologyopen.

[11] Magne F., Gotteland M., Gauthier L., Zazueta A., Pesoa S., Navarrete P., Balamurugan R. (2020). The Firmicutes/Bacteroidetes Ratio: A Relevant Marker of Gut Dysbiosis in Obese Patients?. Nutrients.

[12] Grech A., Collins E.C., Holmes A., Lal R., Duncanson K., Taylor R., Gordon A. (2021). Maternal exposures and the infant gut microbiome: A systematic review with meta-analysis. Gut Microbes.

[13] Wang C., Cui M.L., Wang S.N., Zhu X.P. (2022). Intestinal microbiome and its relationship with necrotizing enterocolitis in very low birth weight preterm infants. Zhonghua er ke za zhi Chin. J. Pediatr..

[14] Zhou Y., Shan G., Sodergren E., Weinstock G., Walker W.A., Gregory K.E. (2015). Longitudinal Analysis of the Premature Infant Intestinal Microbiome Prior to Necrotizing Enterocolitis: A Case-Control Study. PLoS ONE.

[15] Zhao Q., Shi Q., Zhu Q., Hu Y., Zhang X. (2022). A mini-review of advances in intestinal flora and necrotizing enterocolitis. Lett. Appl. Microbiol..

[16] Pammi M., Cope J., Tarr P.I., Warner B.B., Morrow A.L., Mai V., Gregory K.E., Kroll J.S., McMurtry V., Ferris M.J. (2017). Intestinal dysbiosis in preterm infants preceding necrotizing enterocolitis: A systematic review and meta-analysis. Microbiome.

[17] Denning N.-L., Prince J.M. (2018). Neonatal intestinal dysbiosis in necrotizing enterocolitis. Mol. Med..

[18] Neu J., Pammi M. (2018). Necrotizing enterocolitis: The intestinal microbiome, metabolome and inflammatory mediators. Semin. Fetal Neonatal Med..

[19] Yañez C.M., Hernández A.M., Sandoval A.M., Domínguez M.A.M., Muñiz S.A.Z., Gómez J.O.G. (2021). Prevalence of Blastocystis and its association with Firmicutes/Bacteroidetes ratio in clinically healthy and metabolically ill subjects. BMC Microbiol..

[20] Underwood M.A., Sohn K. (2017). The Microbiota of the Extremely Preterm Infant. Clin. Perinatol..

[21] Gomaa E.Z. (2020). Human gut microbiota/microbiome in health and diseases: A review. Antonie Van Leeuwenhoek.

[22] Vandenplas Y., Carnielli V.P., Ksiazyk J., Luna M.S., Migacheva N., Mosselmans J.M., Picaud J.C., Possner M., Singhal A., Wabitsch M. (2020). Factors affecting early-life intestinal microbiota development. Nutrition.

[23] Carlson A.L., Xia K., Azcarate-Peril M.A., Goldman B.D., Ahn M., Styner M.A., Thompson A.L., Geng X., Gilmore J.H., Knickmeyer R.C. (2018). Infant Gut Microbiome Associated With Cognitive Development. Biol. Psychiatry.

[24] Aatsinki A.-K., Lahti L., Uusitupa H.-M., Munukka E., Keskitalo A., Nolvi S., O’Mahony S., Pietilä S., Elo L.L., Eerola E. (2019). Gut microbiota composition is associated with temperament traits in infants. Brain Behav. Immun..

[25] Buffington S.A., Di Prisco G.V., Auchtung T.A., Ajami N.J., Petrosino J.F., Costa-Mattioli M. (2016). Microbial Reconstitution Reverses Maternal Diet-Induced Social and Synaptic Deficits in Offspring. Cell.

[26] Hernández-Martínez C., Canals J., Voltas N., Martín-Luján F., Arija V. (2022). Circulating Levels of Short-Chain Fatty Acids during Pregnancy and Infant Neurodevelopment. Nutrients.

[27] Yang L.L., Millischer V., Rodin S., MacFabe D.F., Villaescusa J.C., Lavebratt C. (2020). Enteric short-chain fatty acids promote proliferation of human neural progenitor cells. J. Neurochem..

[28] Yu L., Zhong X., He Y., Shi Y. (2020). Butyrate, but not propionate, reverses maternal diet-induced neurocognitive deficits in offspring. Pharmacol. Res..

[29] Lyons K.E., Ryan C.A., Dempsey E.M., Ross R.P., Stanton C. (2020). Breast Milk, a Source of Beneficial Microbes and Associated Benefits for Infant Health. Nutrients.

[30] Fernández-Ferreiro A., Formigo-Couceiro F.J., Veiga-Gutierrez R., Maldonado-Lobón J.A., Hermida-Cao A.M., Rodriguez C., Bañuelos O., Olivares M., Blanco-Rojo R. (2022). Effects of *Loigolactobacillus coryniformis* K8 CECT 5711 on the Immune Response of Elderly Subjects to COVID-19 Vaccination: A Randomized Controlled Trial. Nutrients.

[31] Carr L.E., Virmani M.D., Rosa F., Munblit D., Matazel K.S., Elolimy A.A., Yeruva L. (2021). Role of Human Milk Bioactives on Infants’ Gut and Immune Health. Front. Immunol..

[32] Moubareck C. (2021). Human Milk Microbiota and Oligosaccharides: A Glimpse into Benefits, Diversity, and Correlations. Nutrients.

[33] Zwittink R.D., Renes I.B., van Lingen R.A., van Zoeren-Grobben D., Konstanti P., Norbruis O.F., Martin R., Jebbink L.J.M.G., Knol J., Belzer C. (2018). Association between duration of intravenous antibiotic administration and early-life microbiota development in late-preterm infants. Eur. J. Clin. Microbiol. Infect. Dis..

[34] Kim H., Sitarik A.R., Woodcroft K., Johnson C.C., Zoratti E. (2019). Birth Mode, Breastfeeding, Pet Exposure, and Antibiotic Use: Associations With the Gut Microbiome and Sensitization in Children. Curr. Allergy Asthma Rep..

[35] Stearns J.C., Simioni J., Gunn E., McDonald H., Holloway A.C., Thabane L., Mousseau A., Schertzer J.D., Ratcliffe E.M., Rossi L. (2017). Intrapartum antibiotics for GBS prophylaxis alter colonization patterns in the early infant gut microbiome of low risk infants. Sci. Rep..

[36] Ramirez J., Guarner F., Fernandez L.B., Maruy A., Sdepanian V.L., Cohen H. (2020). Antibiotics as Major Disruptors of Gut Microbiota. Front. Cell. Infect. Microbiol..

[37] Aloisio I., Quagliariello A., De Fanti S., Luiselli D., De Filippo C., Albanese D., Corvaglia L.T., Faldella G., Di Gioia D. (2016). Evaluation of the effects of intrapartum antibiotic prophylaxis on newborn intestinal microbiota using a sequencing approach targeted to multi hypervariable 16S rDNA regions. Appl. Microbiol. Biotechnol..

[38] Becattini S., Taur Y., Pamer E.G. (2016). Antibiotic-Induced Changes in the Intestinal Microbiota and Disease. Trends Mol. Med..

[39] Coker M.O., Hoen A.G., Dade E., Lundgren S., Li Z., Wong A.D., Zens M.S., Palys T.J., Morrison H.G., Sogin M.L. (2019). Specific class of intrapartum antibiotics relates to maturation of the infant gut microbiota: A prospective cohort study. BJOG Int. J. Obstet. Gynaecol..

[40] Dierikx T.H., Berkhout D.J.C., Visser L., Benninga M.A., Roeselers G., De Boer N.K.H., De Vries J.I.P., De Meij T.G.J. (2019). The influence of timing of Maternal administration of Antibiotics during cesarean section on the intestinal Microbial colonization in Infants (MAMI-trial): Study protocol for a randomised controlled trial. Trials.

[41] Zou Z.-H., Liu D., Li H.-D., Zhu D.-P., He Y., Hou T., Yu J.-L. (2018). Prenatal and postnatal antibiotic exposure influences the gut microbiota of preterm infants in neonatal intensive care units. Ann. Clin. Microbiol. Antimicrob..

[42] Jokela R., Korpela K., Jian C., Dikareva E., Nikkonen A., Saisto T., Skogberg K., de Vos W.M., Kolho K.-L., Salonen A. (2022). Quantitative insights into effects of intrapartum antibiotics and birth mode on infant gut microbiota in relation to well-being during the first year of life. Gut Microbes.

[43] Gerber J.S., Bryan M., Ross R.K., Daymont C., Parks E.P., Localio A.R., Grundmeier R.W., Stallings V.A., Zaoutis T.E. (2016). Antibiotic Exposure During the First 6 Months of Life and Weight Gain During Childhood. JAMA.

[44] Rood M., Ten Kate L., Boeddha N.P., van‘t Kruys K. (2023). Clinical Characteristics, Transmission Rate and Outcome of Neonates Born to COVID-19-Positive Mothers: A Prospective Case Series From a Resource-Limited Setting. Pediatr. Infect. Dis. J..

[45] Langford B.J., So M., Raybardhan S., Leung V., Westwood D., MacFadden D.R., Soucy J.-P.R., Daneman N. (2020). Bacterial co-infection and secondary infection in patients with COVID-19: A living rapid review and meta-analysis. Clin. Microbiol. Infect..

[46] Lansbury L., Lim B., Baskaran V., Lim W.S. (2020). Co-infections in humans with COVID-19: A systematic review and meta-analysis. J Infect..

[47] Rizvi S.G., Ahammad S.Z. (2022). COVID-19 and antimicrobial resistance: A cross-study. Sci. Total. Environ..

[48] Taylor L. (2021). Covid-19: Antimicrobial misuse in Americas sees drug resistant infections surge, says WHO. BMJ.

[49] Lai C.-C., Chen S.-Y., Ko W.-C., Hsueh P.-R. (2021). Increased antimicrobial resistance during the COVID-19 pandemic. Int. J. Antimicrob. Agents.

[50] Sharma S., Barman P., Joshi S., Preet S., Saini A. (2022). Multidrug resistance crisis during COVID-19 pandemic: Role of anti-microbial peptides as next-generation therapeutics. Colloids Surf. B Biointerfaces.

[51] Köstlbacher S., Collingro A., Halter T., Domman D., Horn M. (2021). Coevolving Plasmids Drive Gene Flow and Genome Plasticity in Host-Associated Intracellular Bacteria. Curr. Biol..

[52] Yu D., Meng X., de Vos W.M., Wu H., Fang X., Maiti A.K. (2021). Implications of Gut Microbiota in Complex Human Diseases. Int. J. Mol. Sci..

[53] Campbell C., Kandalgaonkar M.R., Golonka R.M., Yeoh B.S., Vijay-Kumar M., Saha P. (2023). Crosstalk between Gut Microbiota and Host Immunity: Impact on Inflammation and Immunotherapy. Biomedicines.

[54] Daniel N., Lécuyer E., Chassaing B. (2021). Host/microbiota interactions in health and diseases—Time for mucosal microbiology!. Mucosal Immunol..

[55] Hou K., Wu Z.-X., Chen X.-Y., Wang J.-Q., Zhang D., Xiao C., Zhu D., Koya J.B., Wei L., Li J. (2022). Microbiota in health and diseases. Signal Transduct. Target. Ther..

[56] Agnihotri N., Mohajeri M.H. (2022). Involvement of Intestinal Microbiota in Adult Neurogenesis and the Expression of Brain-Derived Neurotrophic Factor. Int. J. Mol. Sci..

[57] Mitev K., Taleski V. (2019). Association between the Gut Microbiota and Obesity. Open Access Maced. J. Med. Sci..

[58] Ege M.J. (2017). The Hygiene Hypothesis in the Age of the Microbiome. Ann. Am. Thorac. Soc..

[59] Arrieta M.-C., Arévalo A., Stiemsma L., Dimitriu P., Chico M.E., Loor S., Vaca M., Boutin R.C., Morien E., Jin M. (2018). Associations between infant fungal and bacterial dysbiosis and childhood atopic wheeze in a nonindustrialized setting. J. Allergy Clin. Immunol..

[60] Moossavi S., Miliku K., Sepehri S., Khafipour E., Azad M.B. (2018). The Prebiotic and Probiotic Properties of Human Milk: Implications for Infant Immune Development and Pediatric Asthma. Front. Pediatr..

[61] Marzeta C.B., Burgos F., Del Compare M., Gerold I., Tabacco O., Vinderola G. (2022). Approach to probiotics in pediatrics: The role of Lactobacillus rhamnosus GG. Acceso Abiert.

[62] Prame Kumar K., Nicholls A.J., Wong C.H.Y. (2018). Partners in crime: Neutrophils and monocytes/macrophages in inflammation and disease. Cell Tissue Res..

[63] Sproston N.R., Ashworth J.J. (2018). Role of C-Reactive Protein at Sites of Inflammation and Infection. Front. Immunol..

[64] Osredkar J., Kurent T., Fabjan T., Kumer K., Alič E.B., Drobne D. (2021). The comparison of the three assays for determination of fecal calprotectin in inflammatory bowel disease. Biochem. Med..

[65] Kostas A., Siakavellas S.I., Kosmidis C., Takou A., Nikou J., Maropoulos G., Vlachogiannakos J., Papatheodoridis G.V., Papaconstantinou I., Bamias G. (2017). Fecal calprotectin measurement is a marker of short-term clinical outcome and presence of mucosal healing in patients with inflammatory bowel disease. World J. Gastroenterol..

[66] Mari A., Abu Baker F., Mahamid M., Yacoob A., Sbeit W., Khoury T. (2019). Clinical utility of fecal calprotectin: Potential applications beyond inflammatory bowel disease for the primary care physician. Ann. Gastroenterol..

[67] Sturgeon C., Fasano A. (2016). Zonulin, a regulator of epithelial and endothelial barrier functions, and its involvement in chronic inflammatory diseases. Tissue Barriers.

[68] Szymanska E., Wierzbicka A., Dadalski M., Kierkus J. (2021). Fecal Zonulin as a Noninvasive Biomarker of Intestinal Permeability in Pediatric Patients with Inflammatory Bowel Diseases—Correlation with Disease Activity and Fecal Calprotectin. J. Clin. Med..

[69] Caviglia G.P., Rosso C., Ribaldone D.G., Dughera F., Fagoonee S., Astegiano M., Pellicano R. (2019). Physiopathology of intestinal barrier and the role of zonulin. Minerva Biotechnol. Biomol. Res..

[70] Sochaczewska D., Ziętek M., Dołęgowska B., Kordek A., Szczuko M. (2022). Implications of Indirect Biomarkers of Intestinal Permeability in the Stools of Newborns and Infants with Perinatal Risk Factors for Intestinal Colonization Disorders and Infant Feeding Patterns. Nutrients.

[71] Łoniewska B., Węgrzyn D., Adamek K., Kaczmarczyk M., Skonieczna-Żydecka K., Adler G., Jankowska A., Uzar I., Kordek A., Celewicz M. (2019). The Influence of Maternal-Foetal Parameters on Concentrations of Zonulin and Calprotectin in the Blood and Stool of Healthy Newborns during the First Seven Days of Life. An Observational Prospective Cohort Study. J. Clin. Med..

[72] Titus A.S.C.L.S., Vanarsa K., Soomro S., Patel A., Prince J., Kugathasan S., Mohan C. (2023). Resistin, Elastase, and Lactoferrin as Potential Plasma Biomarkers of Pediatric Inflammatory Bowel Disease Based on Comprehensive Proteomic Screens. Mol. Cell. Proteom..

[73] Rodríguez-Benítez M.V., Gámez-Belmonte R., Gil-Campos M., Hernández-Chirlaque C., Bouzas P.R., de Medina F.S., Martínez-Augustin O. (2021). Premature Birth Infants Present Elevated Inflammatory Markers in the Meconium. Front. Pediatr..

[74] Siraki G.A. (2021). The many roles of myeloperoxidase. From inflammation and immunity to biomarkers, drug metabolism and drug discovery. Redox Biol..

[75] Kim S., Kim G.-H. (2017). Roles of claudin-2, ZO-1 and occludin in leaky HK-2 cells. PLoS ONE.

[76] Vancamelbeke M., Vermeire S. (2017). The intestinal barrier: A fundamental role in health and disease. Expert Rev. Gastroenterol. Hepatol..

[77] Kinashi Y., Hase K. (2021). Partners in Leaky Gut Syndrome: Intestinal Dysbiosis and Autoimmunity. Front. Immunol..

[78] Stan T.L., Soylu-Kucharz R., Burleigh S., Prykhodko O., Cao L., Franke N., Sjögren M., Haikal C., Hållenius F., Björkqvist M. (2020). Increased intestinal permeability and gut dysbiosis in the R6/2 mouse model of Huntington’s disease. Sci. Rep..

[79] Gómez-López A. (2019). Microbioma, salud y enfermedad: Probióticos, prebióticos y symbióticos. Biomedica.

[80] Haran J.P., McCormick B.A. (2021). Aging, Frailty, and the Microbiome-How Dysbiosis Influences Human Aging and Disease. Gastroenterology.

